# Corrections of photon beam profiles of small fields measured with ionization chambers using a three‐layer neural network

**DOI:** 10.1002/acm2.13447

**Published:** 2021-10-11

**Authors:** Ann‐Britt Schönfeld, Karl Mund, Guanghua Yan, Andreas Alexander Schönfeld, Hui Khee Looe, Björn Poppe

**Affiliations:** ^1^ University Clinic for Medical Radiation Physics Medical Campus Pius Hospital Carl von Ossietzky University, Oldenburg Germany; ^2^ Department of Radiation Oncology University of Florida Gainesville Florida USA; ^3^ Sun Nuclear Corp. Melbourne Florida USA

**Keywords:** deconvolution, ionization chamber, neural network, small field dosimetry, volume‐averaging effect

## Abstract

The purpose of this work is to study the feasibility of photon beam profile deconvolution using a feedforward neural network (NN) in very small fields (down to 0.56 × 0.56 cm^2^). The method's independence of the delivery and scanning system is also investigated. Lateral beam profiles of photon fields between 0.56 × 0.56 cm^2^ and 4.03 × 4.03 cm^2^ were collected on a Siemens Artiste linear accelerator. Three scanning ionization chambers (SNC 125c, PTW 31021, and PTW 31022) of sensitive volumes ranging from 0.016 cm^3^ to 0.108 cm^3^ were used with a PTW MP3 water phantom. A reference dataset was also collected with a PTW 60019 microDiamond detector to train and test individual NNs for each ionization chamber. Further testing of the trained NNs was performed with additional test data collected on an Elekta Synergy linear accelerator using a Sun Nuclear 3D Scanner. The results were evaluated with a 1D gamma analysis (0.5 mm/0.5%). After the deconvolution, the gamma passing rates increased from 54.79% to 99.58% for the SNC 125c, from 57.09% to 99.83% for the PTW 31021, and from 91.03% to 96.36% for the PTW 31022. The delivery system, the scanning system, the scanning mode (continuous vs. step‐by‐step), and the electrometer had no significant influence on the results. This study successfully demonstrated the feasibility of using NN to correct the beam profiles of very small photon fields collected with ionization chambers of various sizes. Its independence of the delivery and scanning system was also shown.

## 1 INTRODUCTION

The wide availability of linear accelerators capable of delivering stereotactic radiosurgery and volumetric‐modulated arc therapy (VMAT) with a high‐modulation complexity has led to an escalated use of small radiation fields in radiotherapy. It has been well documented that small radiation fields are metrologically more challenging than conventional large fields. The challenges arise from the partial occlusion of the primary photon source by the collimation system and the loss of lateral charged particle equilibrium. Additionally, in non‐equilibrium conditions of small radiation fields, the perturbations caused by the detector itself can significantly impact the measurement.

Accurate dose profile measurements play an essential role in linear accelerator commissioning, quality assurance, and patient plan verification measurements. Even though ionization chambers are the gold standard for dose profile measurements due to their minimal energy dependence, excellent dose linearity, long‐term stability, as well as the thorough theoretical considerations, their comparably large size usually leads to a significant volume‐averaging effect (VAE). In small fields, the VAE, in combination with the density effect, causes an underestimation of the dose maximum and a significant broadening of the penumbra region.

Both TRS 483^1^ and DIN 6809‐8^2^ suggest to perform corrections of lateral beam profiles measured with small ionization chambers to eliminate the impact of the detector's VAE and density effect. This correction can be performed based on the mathematical description of the measurement process, where the measured signal profile is the result of the convolution of the dose profile and the detector's lateral dose‐response function.^3^, ^4^, ^5^ Several deconvolution techniques using numerical^4^, ^6^, ^7^ or analytical^8^, ^9^, ^10^, ^11^ approaches have been published to demonstrate the underlying mathematical model and the feasibility of the respective methods to obtain the unperturbed dose profiles. Nevertheless, most studies investigated relatively large radiation beams with field sizes above 2 × 2 cm^2^, where the perturbations are only limited to the field borders. In small or very small fields, the VAE and density effect may cause signal perturbation along the whole profile due to overlapping penumbra regions. In these cases, where the small field perturbations are most prominent and the associated dosimetry is most challenging, studies on the corrections of these profile measurements are still scarce. A major drawback of the analytical and numerical deconvolution techniques is the required knowledge of the detector‐specific lateral dose‐response function K(x). Moreover, deconvolution techniques relying on Fourier transformations are sensitive to high‐frequency noises in the measurement signal.

Liu et al.^12^ proposed a deconvolution method using a three‐layer neural network (NN). Its feasibility has been shown for 6 MV photon fields between 2 × 2 cm^2^ and 10 × 10 cm^2^. Mund et al.^13^ extended the study to flattening‐filter‐free (FFF) beams and evaluated the performance of energy‐specific and combined networks (one network trained for multiple beam energies) for photon fields with field size ≥ 2 × 2 cm^2^. The applicability of the NN on small field sizes (< 2 × 2 cm^2^) has not been studied.

Therefore, the aim of this work was to study the applicability of an artificial NN to correct beam profiles of small photon beams (from 0.56 × 0.56 cm^2^ to 4.03 × 4.03 cm^2^) measured with ionization chambers of various sensitive volumes. This work also demonstrated the transferability of the pre‐trained NN model from one linear accelerator/scanning system to another for the first time. Furthermore, we compared the NN approach with other numerical and analytical deconvolution methods in the context of very small photon fields.

## 2 METHODS

### 2.1 Measurement setup

Two datasets were collected for this study. The first dataset was collected on a Siemens Artiste linear accelerator (Siemens, Munich, Germany) with three different ionization chambers and a microDiamond detector (Table 1). Lateral beam profiles of 6 MV photon fields were measured with a PTW MP3‐M water phantom (PTW Freiburg, Germany). The measurements were performed at a 90 cm source‐to‐surface distance (SSD) for fields of [0.56 × 0.56, 0.62 × 0.62, 0.70 × 0.70, 0.77 × 0.77, 0.85 × 0.85, 0.93 × 0.93, 1.01 × 1.01, 1.10 × 1.10, 1.29 × 1.29, 1.58 × 1.58, 1.77 × 1.77, 2.07 × 2.07, 2.56 × 2.56, 3.05 × 3.05, 3.54 × 3.54, 4.03 × 4.03] cm^2^. The profiles at depths of 1.5 cm, 5 cm, 10 cm, and 20 cm were collected in a step‐by‐step mode with a 0.03 cm step size. The PTW Trufix System was used to move the effective point of measurement of the ionization chamber to the scanning depth. The Center Check tool of the Mephysto software (PTW) was used to position the detector at the dose maximum each time the field size or the detector was changed.

**TABLE 1:** Physical properties of the detectors used for the measurements

	**Diameter of sensitive volume (mm)**	**Length of sensitive volume (mm)**	**Sensitive volume**	**Nominal response (nC/Gy)**	**Operating voltage (V)**
SNC 125c Scanning	4.75	7.05	0.108 cm^3^	3.4	300
PTW 31021 Semiflex 3D	4.8	4.8	0.070 cm^3^	2.0	400
PTW 31022 PinPoint 3D	2.9	2.9	0.016 cm^3^	0.4	300
PTW 60019 microDiamond	2.2	0.002	0.004 mm^3^	1.0	0
SNC EDGE	0.8	0.03	0.019 mm^3^	32	0

Table 1 lists the physical properties of the detectors used in this study. The integration time was 0.1 s for all the ionization chambers and 0.5 s for the microDiamond detector to ensure a sufficient signal‐to‐noise ratio. Following the recommendations of the TRS 483 report,^1^ all ionization chambers were oriented radially, i.e., the chamber's axis was perpendicular to the beam's axis, and the scans were performed along the radial direction of the chambers. The microDiamond detector was chosen as the reference detector due to its advantageous dosimetric properties.^14^, ^15^ Contrary to air‐filled chambers, where the density perturbation is causing further beam broadening, the enhanced density of the microDiamond components is causing the opposite effect, i.e., penumbra steepening, and hence compensating largely the VAE. The detector was placed axially such that the detector's axis aligned parallelly to the beam's central axis.

A second dataset was recorded on an Elekta Synergy linear accelerator (Elekta AB, Stockholm, Sweden) to investigate the NN's independence of the beam delivery system and the dosimetry equipment. Both the SNC 125c and the SNC EDGE detector (Sun Nuclear Corp., Melbourne, USA) were used with a SNC 3D Scanner water phantom (Sun Nuclear Corp., Melbourne, USA) in the continuous scanning mode. The NN trained for the SNC 125c with the first dataset was then directly applied to deconvolve the profiles collected with the SNC 125c in this dataset. The deconvolved profiles were compared with the profiles measured with the SNC EDGE detector. Table 2 shows the main differences between this work and the previous studies.

**TABLE 2:** Main characteristics of NN deconvolution studies

	**Liu et al**.^12^	**Mund et al**.^13^	**This study**
Nominal field sizes	2 × 2 cm^2^ – 10 × 10 cm^2^	2 × 2 cm^2^ – 10 × 10 cm^2^	0.3 × 0.3 – 4 × 4 cm^2^
Photon beams	6 MV	6 MV, 6 FFF, 10 FFF	6 MV
Linear accelerator	Elekta Versa HD	Elekta Versa HD	Siemens Artiste, Elekta Synergy
Ionization chambers	IBA CC13	CC04, CC13, FC65‐P (all IBA)	SNC 125c, PTW 31021, PTW 31022
Reference detector	SNC EDGE	SNC EDGE	PTW 60019 microDiamond
Major findings	Feasibility of NN method	Various ICs, different energies and modalities, separate and combined NNs	Various ICs, feasibility for small field application, independent test data from different linear accelerator/equipment

### 2.2 Neural network deconvolution method

The convolution model describes the measurement process with a non‐ideal detector. The detector's perturbation can be characterized by the lateral dose‐response function K(x) which is a convolution kernel connecting the dose profile D(x) with the measured profile M(x).^3^, ^4^, ^5^ The measured profile can thereby be written as:

(1) $$M\left(x\right)=K\left(x\right)*D\left(x\right),$$

where * represents the convolution operator. Therefore, the dose profile can be derived by deconvolution, i.e.,

(2) $$D\left(x\right)=K^{-1}\;\left(x\right)*M\left(x\right),$$

where $K^{-1}\left(x\right)$ is the deconvolution kernel.^3^, ^4^, ^5^

In this study, we employed the feedforward three‐layer NN proposed by Liu et al.^12^ to solve the deconvolution problem. The three‐layer structure consists of an input layer, a hidden layer, and an output layer. A sliding window extracts input data $s_{j}$ from the measured profiles for the NN. The length of the sliding window $L_{\mathrm{sw}}$ corresponds to the number of input nodes. The size of the hidden layer is the number of hidden neurons ($N_{\mathrm{hn}}$) that are fully connected to the input nodes and the single node of the output layer. The deconvolved value is calculated by the output node at the center of the sliding window. As the window moves over the entire measured profile, the deconvolved profile is obtained in a pointwise fashion.

The dataset was divided into three sub‐datasets to train, validate, and test the NN (Table 3). A separate NN model was trained for each ionization chamber shown in Table 1. A parametric sweeping method was used to find the optimal combination of $L_{\mathrm{sw}}$ and $N_{\mathrm{hn}}$. The ideal combination of $L_{\mathrm{sw}}$ and $N_{\mathrm{hn}}$ was chosen based on the performance of the NN on training and validation data, which was evaluated by a 1D gamma analysis^16^, ^17^ with a 0.5 mm/1% (global) criterion and a 10% dose threshold (TH).

**TABLE 3:** Separation of training, validation, and test datasets

	**Training dataset**	**Validation dataset**	**Test dataset**
**Dosimetric** (nominal) field sizes (cm^2^)	**0.56 × 0.56** (0.3 × 0.3) **0.70 × 0.70** (0.5 × 0.5) **0.85 × 0.85** (0.7 × 0.7) **1.10 × 1.10** (1.0 × 1.0) **1.58 × 1.58** (1.5 × 1.5) **2.07 × 2.07** (2.0 × 2.0) **3.05 × 3.05** (3.0 × 3.0) **4.03 × 4.03** (4.0 × 4.0)	**0.56 × 0.56** (0.3 × 0.3) **0.70 × 0.70** (0.5 × 0.5) **0.85 × 0.85** (0.7 × 0.7) **1.10 × 1.10** (1.0 × 1.0) **1.58 × 1.58** (1.5 × 1.5) **2.07 × 2.07** (2.0 × 2.0) **3.05 × 3.05** (3.0 × 3.0) **4.03 × 4.03** (4.0 × 4.0)	**0.62 × 0.62** (0.4 × 0.4) **0.77 × 0.77** (0.6 × 0.6) **0.93 × 0.93** (0.8 × 0.8) **1.01 × 1.01** (0.9 × 0.9) **1.29 × 1.29** (1.2 × 1.2) **1.77 × 1.77** (1.7 × 1.7) **2.56 × 2.56** (2.5 × 2.5) **3.54 × 3.54** (3.5 × 3.5)
Depth (cm)	1.5 10 20	5.0	1.5 5.0 10 20

Bold values correspond to the dosimetric field sizes, nominal field sizes are shown in brackets.

Prior to training the networks, all profiles were normalized to maximum and fitted with the Matlab Curve Fitting Toolbox (Matlab R2019a; The MathWorks, Inc., Natick, MA, USA) using a smoothing spline with a smoothing parameter of 0.95. The deconvolved profiles were compared with the reference profiles using a 1D gamma analysis (0.5 mm/0.5% global) with a 5% dose TH. Additionally, the penumbra width difference (PWD) between the deconvolved and reference profiles was evaluated as

(3) $$\mathrm{PWD}=\left|W-W_{\mathrm{ref}}\right|,$$

where the penumbra width W is the distance between the 20% and 80% points of the profile and $W_{\mathrm{ref}}$ is the penumbra width of the reference profile.

### 2.3 Comparison with other deconvolution methods

The NN approach was compared with three commonly used deconvolution techniques. One of these is based on the convolution theorem that governs Equation (1)^6^, ^7^ using the Fourier transform. The second technique is an iterative method (van Cittert^18^) already applied previously in the field of medical physics^5^, ^19^, ^20^ ^,^ ^21^, ^22^, ^23^. The third approach is an analytical deconvolution method as described by Ulrichs et al.^11^ that generalizes the earlier works of Cho et al.,^24^ García‐Vicente et al.,^8^ Djouguela et al.,^25^ Yan et al.,^10^ and Looe et al.^3^ Contrary to the NN approach, it is noteworthy that all these three approaches require the knowledge of K(x). The comparison was performed exemplarily for the Semiflex 3D 31021 using a Gaussian‐shaped K(x) with a standard deviation of $\sigma_{K}$ = 2.1 mm.^26^

## 3 RESULTS

The parametric sweeping method determined that the optimal combination of parameters $L_{\mathrm{sw}}$ and $N_{\mathrm{hn}}$ were $L_{\mathrm{sw}}=\;9$ (2.7 mm) and $N_{\mathrm{hn}}=\;9$ for all studied ionization chambers. Figure 1 shows various examples of the beam profiles measured with the SNC 125c at 10 cm depth, and the deconvolved profiles. The ionization chamber's volume effect on the measured profiles is clearly visible. The deconvolved profiles have no such effect and resemble closely the reference profiles measured with the microDiamond detector. The bottom plot in each panel of Figure 1 shows the comparison between the deconvolved profile and the reference profile in terms of the computed gamma indices (0.5 mm/0.5%; TH 5%).

**FIGURE 1:**
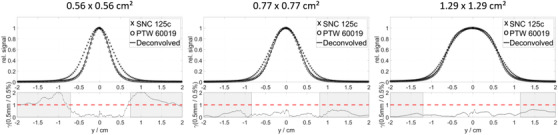
Deconvolution results with the NN method for the SNC 125c for selected fields at 10 cm depth. The bottom plot shows the result of the gamma analysis (0.5 mm/0.5%; TH 5%) comparing the deconvolved profile with the reference profile. The shaded regions indicate the part of the profiles, where the intensity is below the 5% threshold (TH)

Figure 2a summarizes the results from the 1D gamma analysis of all measured profiles for the SNC 125c. Before deconvolution, the gamma index distribution is spread out toward larger values (Figure 2a). Figure 2b shows the gamma index histogram after the NN deconvolution, where around 90% of the data points passed with a gamma index smaller than 0.5, indicating the good performance of the NN. Overall, the deconvolution process increases the gamma passing rates for all deconvolved profiles from 54.79% to 99.58% for the SNC 125c, from 57.09% to 99.83% for the PTW 31021, and from 91.03% to 96.36% for the PTW 31022.

**FIGURE 2:**
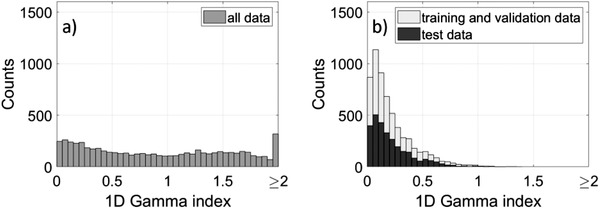
Histograms of the 1D gamma indices for beam profiles measured with the SNC 125c before (a) and after (b) the NN deconvolution. The criterion was 0.5 mm and 0.5% with a 5% threshold

Figure 3 shows the PWDs for the SNC 125c before (labeled as “IC measurement”) and after the deconvolution (labeled as “deconvolved”). Before the deconvolution, profile broadening caused by the chamber's VAE and density effect resulted in larger penumbra width of the measured profiles. Therefore, the calculated PWDs are systematically larger than zero but the magnitude decreases with decreasing chamber's volume (not shown here). These discrepancies have been eliminated by the NN deconvolution, so that the PWDs of the deconvolved profiles are reduced to almost 0 mm. The fluctuation of these PWDs is slightly enhanced by the deconvolution process and ranges from ±0.1 mm (PTW 31022) to ±0.4 mm (SNC 125c).

**FIGURE 3:**
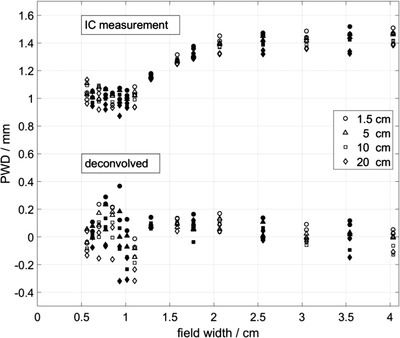
The PWDs of the beam profiles collected with the SNC 125c before (“IC measurement”) and after the NN deconvolution (“deconvolved”). The shapes of the symbols represent different measurement depths. The open and filled symbols indicate training/validation and test data, respectively

Figure 4 shows the results of applying the NN directly on the second dataset for field sizes 0.59 × 0.59 cm^2^, 1.02 × 1.02 cm^2^ and 1.47 × 1.47 cm^2^ at 10 cm depth. The profiles collected with the SNC EDGE detector are used as references. The overall gamma passing rate increases from 39.34% before deconvolution to 77.90% after deconvolution.

**FIGURE 4:**
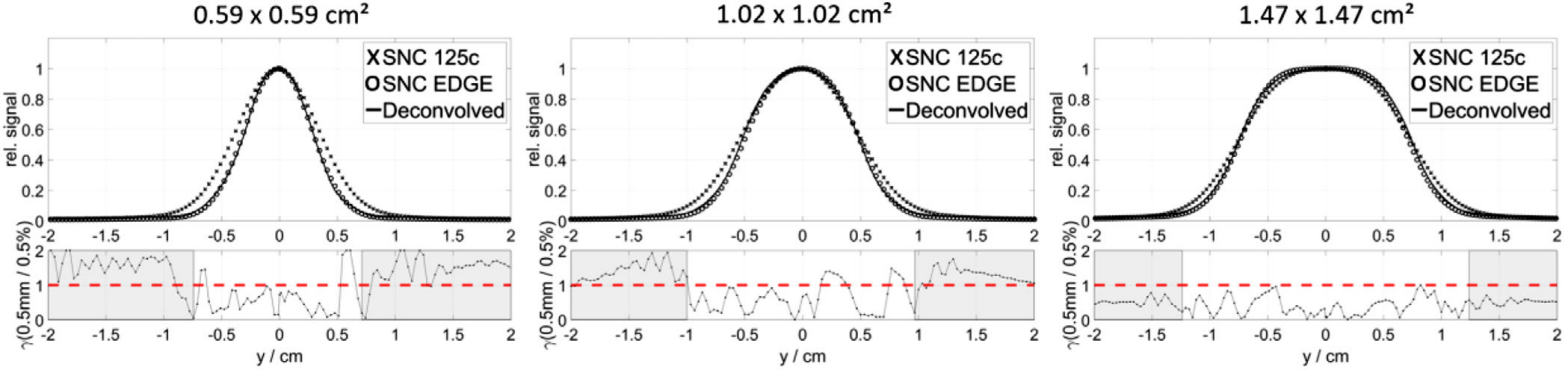
Deconvolution results with the pre‐trained NN using the Siemens Artiste training dataset for the SNC 125c for selected fields at 10 cm depth. The test dataset was acquired at an Elekta Synergy linear accelerator in a Sun Nuclear 3D Scanner water phantom. The bottom plot shows the result of the gamma analysis (0.5 mm/0.5%; TH 5%) comparing the deconvolved profile with the reference profile. The shaded regions indicate the part of the profiles, where the intensity is below the 5% threshold (TH)

Figure 5 shows the comparison between the NN approach and the three other deconvolution techniques. As examples, beam profiles for field sizes 0.62 × 0.62 cm^2^ (left) and 3.54 × 3.54 cm^2^ (right) measured with the PTW 31021 are shown. For the Fourier deconvolution technique, field size‐dependent cutoff‐frequencies were chosen after the Fourier transforms (0.21/mm for the smaller field and 0.09/mm for the larger field). Attention was placed to remove as much high‐frequency noise as possible in the signals but to minimize the truncation of real signal information. Nevertheless, the Fourier deconvolution technique still shows the largest deviation, where further optimization of the cut‐off frequencies has not resulted in significant improvements. The results from the iterative and analytical approaches are very similar, where the deconvolved profiles show a steeper gradient in the outer field region than the reference profile, especially for the smaller field size (Figure 5, left). The discrepancies in these numerical/analytical methods may be partly attributed to the use of a Gaussian approximation of the detector response function. In reality, the detector response function could be a lot more complex.^4^, ^14^ Furthermore, a small fluctuation in the result could be observed using the iterative method due to the noise.

**FIGURE 5:**
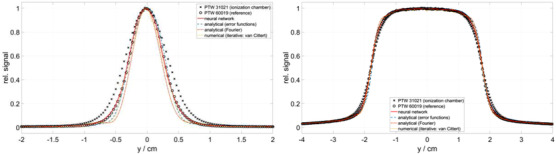
Comparison between the NN approach and three analytical/numerical deconvolution methods. The profiles were measured with the PTW 31021 for a 0.62 × 0.62 cm^2^ field (left) and a 3.54 × 3.54 cm^2^ field (right). In the analytical/numerical methods, the lateral detector response function was approximated with a Gaussian function with a standard deviation of $\sigma_{K}$ = 2.1 mm

## 4 DISCUSSION

Building upon the works of Mund et al.^13^ and Liu et al.,^12^ we demonstrated the feasibility of using the NN deconvolution method on very small photon fields. The sliding window length $L_{\mathrm{sw}}$ and the number of hidden neurons $N_{\mathrm{hn}}$, were reoptimized for these fields. Liu et al.^12^ suggested that $L_{\mathrm{sw}}$ is related to the physical dimensions of the chamber's sensitive volume. For the determination of $L_{\mathrm{sw}}$, they proposed to consider the chamber's diameter as well as the spread of the detector response function. Additionally, for small fields, we found that $L_{\mathrm{sw}}$ should not be larger than the smallest dosimetric field width used for training (0.56 cm in this study) to avoid artifacts in the deconvolved profiles.

Our results demonstrated the capability of the NN method to correct for perturbation effects caused by VAE and the density effect of all ionization chambers at all the studied field sizes. As seen in Figure 3, the PWD before the deconvolution increases with the field size up to about 2 × 2 cm^2^. The PWD remains constant within the uncertainty at larger field sizes, which was tested for fields up to a field size of 20 × 20 cm^2^. The field size dependence of the penumbra width *W* in smaller field sizes can be traced back to the small field phenomenon of apparent field widening.^1^ Furthermore, the PWDs of the ionization chamber measurements also decrease with the chamber size due to a declining volume effect. Such unique features of small fields should be adequately represented in the training dataset. Consequently, a combined NN involving field sizes up to 20 × 20 cm^2^ yielded worse deconvolution results for the small field profiles. Therefore, this study included photon fields up to 4 × 4 cm^2^, which corresponds to the smallest reasonable field size for conventional reference dosimetry according to DIN 6809‐8.^2^

The choice of the reference detector influences directly the results obtained using the deconvolution method. The previous studies^12^, ^13^ mostly used silicon diodes as a reference, but these are subject to the density effect^15^, ^27^ in small fields and additionally, in larger depths or field sizes, to energy dependence. In this study, the microDiamond detector with more advantageous properties^14^, ^15^, ^28^ has been chosen as the reference detector to train the NN. In the second independent test dataset acquired at a different linear accelerator, the deconvolution results have been compared to that measured using a silicon diode due to technical incompatibilities of the microDiamond with equipment from another vendor. Nevertheless, the results in Figure 4 demonstrated that deconvolution using the pre‐trained model delivers clinically acceptable results that agree with the diode measurements.

The results from the three analytical and numerical deconvolution techniques evaluated in this work are subject to the choice of K(x). Based on the results in Figure 5, reducing the standard deviation $\sigma_{K}$ of the Gaussian approximation of K(x) has resulted in better agreement to the microDiamond reference profile. It is noteworthy that the $\sigma_{K}$ = 2.1 mm used in this work has been determined by Delfs et al.^26^ using a silicon diode as the reference detector, which is known to result in a signal profile that is steeper than the dose profile due to the associated density effect. Furthermore, the NN method also possesses the advantage of being more robust to noise than the Fourier or iterative deconvolution.

## AUTHOR CONTRIBUTION STATEMENT

Ann‐Britt Schönfeld: Conception and design of the work; acquisition, analysis, and interpretation of data for the work; drafting the work; final approval of the version to be published; agreement to be accountable for all aspects of the work in ensuring that questions related to the accuracy or integrity of any part of the work are appropriately investigated and resolved.

Karl Mund: Analysis of data for the work; revising the work critically for important intellectual content; final approval of the version to be published; agreement to be accountable for all aspects of the work in ensuring that questions related to the accuracy or integrity of any part of the work are appropriately investigated and resolved.

Guanghua Yan: Conception and design of the work; interpretation of data for the work; revising the work critically for important intellectual content; final approval of the version to be published; agreement to be accountable for all aspects of the work in ensuring that questions related to the accuracy or integrity of any part of the work are appropriately investigated and resolved.

Andreas Alexander Schönfeld: Acquisition, analysis, and interpretation of data for the work; revising the work critically for important intellectual content; final approval of the version to be published; agreement to be accountable for all aspects of the work in ensuring that questions related to the accuracy or integrity of any part of the work are appropriately investigated and resolved.

Hui Khee Looe: Design of the work; interpretation of data for the work; drafting the work and revising it critically for important intellectual content; final approval of the version to be published; agreement to be accountable for all aspects of the work in ensuring that questions related to the accuracy or integrity of any part of the work are appropriately investigated and resolved.

Björn Poppe: Conception and design of the work; interpretation of data for the work; revising the work critically for important intellectual content; final approval of the version to be published; agreement to be accountable for all aspects of the work in ensuring that questions related to the accuracy or integrity of any part of the work are appropriately investigated and resolved.

## CONFLICT OF INTEREST

One of the authors, Andreas Schönfeld, is a Sun Nuclear employee. The authors declare that they have no known competing financial interests or personal relationships that could have appeared to influence the work reported in this paper.
